# Prediction Models in Veterinary and Human Epidemiology: Our Experience With Modeling Sars-CoV-2 Spread

**DOI:** 10.3389/fvets.2020.00513

**Published:** 2020-08-25

**Authors:** Tariq Halasa, Kaare Græsbøll, Matthew Denwood, Lasse Engbo Christensen, Carsten Kirkeby

**Affiliations:** ^1^Section for Animal Welfare and Disease Control, Institute of Veterinary and Animal Sciences, Faculty of Medical and Health Sciences, University of Copenhagen, Frederiksberg, Denmark; ^2^Department of Applied Mathematics and Computer Sciences, Technical University of Denmark, Lyngby, Denmark

**Keywords:** modeling, infectious, disease, spread, COVID-19

## Abstract

The worldwide outbreak of Sars-CoV-2 resulted in modelers from diverse fields being called upon to help predict the spread of the disease, resulting in many new collaborations between different institutions. We here present our experience with bringing our skills as veterinary disease modelers to bear on the field of human epidemiology, building models as tools for decision makers, and bridging the gap between the medical and veterinary fields. We describe and compare the key steps taken in modeling the Sars-CoV-2 outbreak: criteria for model choices, model structure, contact structure between individuals, transmission parameters, data availability, model validation, and disease management. Finally, we address how to improve on the contingency infrastructure available for Sars-CoV-2.

## Introduction

Infectious diseases are a constant threat for public health and consequently also the economy. Although hygiene measures have been well-established and efficient prevention and control measures such as vaccines have been developed for many diseases, only one human disease (smallpox) and one animal disease (Rinderpest) have been eradicated (1). On the other hand, new diseases are emerging and re-emerging in several parts of the world in both humans (e.g., COVID-19) and animals [e.g., African swine fever (ASF) ]. It is therefore important to have consistent and effective systems for rapid and successful control of infectious diseases of both humans and animals. Models of infectious diseases have been used for many years to understand the dynamics of these diseases and to support decision making, and are used in both animal and human populations (2, 3). There is a large overlap with regard to methodology, procedures, and general epidemiological considerations when modeling infectious diseases of animals and humans. The development of models in both contexts is also similarly challenged by several factors such as the availability of data, understanding of the disease and host behavior, and external factors such as the environment.

At the start of the SARS-CoV-2 outbreak in Denmark, an expert group of modelers was established to develop models to predict the course of the epidemic. The authors were part of this group due to their previous experience with modeling disease spread mostly within the veterinary field. In this study, we discuss and compare the challenges for infectious disease prediction models of animal and human populations based on our experience in modeling infectious diseases in animals [e.g., foot-and-mouth disease (FMD), ASF, and bluetongue virus (BTV) ] and our recent experience of modeling the spread of SARS-CoV-2 in humans.

## Choice of Modeling Method

Several modeling methods can be used to mimic the spread of infectious diseases, depending on the disease itself, available data, the need for details, and the purpose of the model (2, 4). Traditionally, ordinary differential equation (ODE) models have been popular, but with increasing computational power, agent-based models (ABMs) that can include higher levels of detail are increasingly being used (4). The purpose of the model is key to the choice of model.

Models are simple representations of real-life systems. In order to be able to build a model that properly represents a given system, it is necessary to have key knowledge in place: (1) a fairly good understanding of how the disease is spread (or knowledge of similar diseases, as for instance for SARS in relation to COVID-19); (2) background data on the host population (demography, density, etc.); and (3) data on the behavior of the host population (mixing patterns). There are two main phases of required models in an outbreak situation for a new disease like COVID-19. During the initial phase where a lockdown of large parts of society is implemented, it is important to have one or more models that can: (1) include the available number of parameters, which are often minimal in number due to the lack of necessary data at the early stage of the epidemic, and (2) run reasonably fast, in order to provide timely predictions on a national/regional level where large number of individuals may be involved. The purpose of modeling in this phase is to evaluate the current (lockdown) situation. In the second phase, where the focus regarding Sars-CoV-2 has been on how to reopen society, it is also important (1) that it is relatively easy to adjust the models to include newly arising information during the outbreak and (2) that the models are flexible and detailed enough to include information on the relevant parts of society.

During the 2001 FMD epidemic in the UK, an ABM with the farm as the modeling unit was used to advise the authorities on the control of the disease (5). Similarly, for the first BTV outbreaks in northern Europe in 2006, models were used to inform authorities on how to react with regard to early warning, mitigation of impact, vaccinating animals, and testing for freedom of disease (6, 7). Another example is the ASF virus genotype II that has persisted in Europe since 2007 and spread to other parts of the world (8). An ABM for the spread of ASF within wild boar populations has been used to advise the European authorities in the control of the disease (9, 10).

In the current Sars-CoV-19 pandemic, many simulation models have been developed, including both ODE and ABM models, of which some have been used to advise authorities. For instance, an ABM was used in the UK to guide the lockdown of the country (11). In the USA, several models were developed and used by the CDC individually or as ensemble modeling to predict the spread of Sars-CoV-19 on a state or country level (12). In Sweden, an ODE model has been used to advise the authorities during the epidemic (13), while a stochastic meta-population model was used in Norway (14). In Denmark, an ODE model was used to advise the authorities (https://github.com/laecdtu/C19DK) and qualitatively supported by an ABM. For previous human epidemics, such as measles, SARS, and influenza, ABM, and ODE models were developed to study disease dynamics and/or guide the control of the epidemics [see details in a review (3)].

## Data on Contact Structure

One of the main challenges in modeling disease spread is identifying and obtaining data on contact structure between the modeled units (e.g., individuals or farms), when heterogeneity is considered. In the veterinary field, the spread of a disease is usually modeled either based on physical contacts between the modeled units (15) or using distance-based kernels (5). In the models that simulate the spread of diseases in the veterinary field using explicit contacts, the spread is driven by contacts between farms via animal movements, indirect contacts (e.g., veterinarians and vehicles), and/or vectors (midges for BTV, air for FMD, and wild boar for ASF). Several countries maintain registers for animal movements between herds, allowing explicit modeling of disease spread between herds (16). Data on indirect contacts is available based on questionnaires and field studies (17). For diseases that spread via vectors, data are provided via experiments and field studies (18–21). For airborne spread, meteorological data have been used to study the spread of FMD (22).

Because humans can normally move freely, while livestock populations are restricted to their farms, humans are more heterogeneous in their activities and contact patterns. Modeling this heterogeneity is therefore important to mimic disease spread correctly. We found few comprehensive studies quantifying contacts and contact patterns between individuals (23–26). These contacts formed the backbone for modeling the spread of Sars-CoV-2 in several models such as [https://github.com/laecdtu/C19DK; (27–29)].

## Data on Disease Stages and Transmission

In the veterinary field, data on the manifestation and stages of infectious diseases within an individual animal and the transmission between individual animals are normally collected based on highly controlled experimental studies (30–32). Such studies are necessary in order to understand and quantify transmission and hence reliably use the data in models of disease spread and control.

In the current Sars-CoV-19 pandemic, data from previous epidemics with other similar viruses such as SARS and influenza were used to parametrize models published at earlier stages (33). Later on, data specifically about Sars-CoV-2 became available from multiple sources (patients, contact tracing, special situations such as cruise ships) allowing the estimation of necessary information regarding disease stages, manifestation, and transmission potential between individuals (34–37). Nevertheless, important information such as proportion of asymptomatic cases, infectiousness and susceptibility of children and their role in disease spread, and the role of superspreaders and superspreading events is yet to be unraveled.

## Data for Modeling and Validation

A general aspect when modeling infectious diseases in real time is fitting models to the available disease occurrence data. For instance, during the 2001 FMD epidemic in the UK, infection spread was modeled by creating a spread kernel using the observed outbreak data (5). Similarly, the spread of ASF within wild boar was simulated by fitting the model to observed data (10). For BTV, the spread in northern Europe has often been modeled using dispersal kernels capturing the vectors being spread in up- and downwind movements (6, 38, 39).

For the current COVID-19 epidemic, several models used to advise the authorities have relied on calibration to hospitalization data rather than the number of test-positive individuals because the latter is known to vary according to changes in testing strategy during the outbreak [https://github.com/laecdtu/C19DK, (13, 14, 40)]. Although this approach is certainly better than the alternatives, it is not without potential pitfalls. During the beginning of the Sars-CoV-2 outbreak in Denmark, substantial technical issues were encountered due to the lack of automated systems for reporting patient numbers. There are also issues around the definitions of “hospitalized due to COVID-19” vs. “hospitalized with COVID-19,” i.e., there exists an unknown number of test-positive patients who have been hospitalized for reasons completely separate from Sars-CoV-19 but happen to be concurrently infected—should these be included in the counts? Given the gradual shift in emphasis from targeted testing toward blanket testing of hospitalized patients, this has the potential to introduce a temporally inconsistent bias in the data from the gradual inclusion of more and more “tangential cases” over time. Put together, these issues pose a substantial challenge for the prediction models, which ideally should be mitigated by including more rigorous randomized testing of individuals to provide an unbiased estimate of the proportion of people that have been infected.

Disease spread models are often only verified to the extent of ensuring that the code does what is intended. Validation of disease spread models is quite challenging due to a lack of comprehensive data for validation and impossible in the case of Sars-CoV-2 models for now. Models developed for specific epidemics may be fitted based on the epidemic data. This does not preclude the fact that such models should also be validated, as they include several parameters that are not necessarily obtained from that specific epidemic.

## Disease Management During An Outbreak

In the veterinary field, the success of disease management in case of an outbreak is highly variable depending on several factors, including the extent of disease spread when the disease is discovered; the severity of the disease; the infectiousness of the virus; the density of the population; the speed of application of control measures; the compliance of animal owners; and the involvement of external factors such as vectors, climate, and/or environmental reservoirs. For instance, the 2001 FMD epidemic in the UK took more than a year to control and spread to surrounding countries such as Ireland, Belgium, and the Netherlands (41). Since the introduction of ASF to Europe in 2007, it has been spreading in several parts of the continent as well as in Southeast Asia (8). Recurrent BTV epidemics have occurred in Europe during the past 15 years affecting several countries (42). The control measures that are normally implemented for outbreaks of these diseases (FMD, ASF, and BTV) may vary from one disease to another, but generally, they include a depopulation of the affected herds followed by cleaning and disinfection, surveillance of neighboring herds, and tracing of contacts. Vaccination may be an option when a vaccine is available, as in the case of BTV (43) and FMD (44).

Since the emergence of reports from Wuhan on the spread of a peculiar disease in late 2019 (44), the disease spread to many countries and continents, leading to a pandemic with devastating economic impact (45). In middle March, Europe was declared the epicenter of the disease (46). The management of the disease in Europe varied from one country to another but was characterized by implementing a lockdown, which varied in the speed and degree of its implementation following increase in hospitalization cases. Some countries such as Denmark quickly implemented a partial countrywide lockdown, while Sweden kept several activities running, including schools, restaurants, and bars (47). These diverging strategies have led them along different paths during the epidemic. Testing, contact tracing, and isolation are measures that were recommended by the World Health Organization (48), and peers emphasized the importance of these measures later when the number of cases is low, in order to cut the transmission chain (46).

## Contingency and Preparedness Plans

Detailed and strict guidelines have been set for the control of highly infectious diseases in the veterinary field. For instance, the EU set clear guidelines for the control of FMD, ASF, and BTV in domestic livestock populations (49–51). The member states must follow these guidelines once the disease is detected in the country and demonstrate preparedness and control plans to prevent onward transmission. Furthermore, regular simulation exercises and assessment of logistic and laboratory capacities must be conducted (15, 52, 53).

The current Sars-CoV-19 epidemic has proven the lack of preparedness of many countries to manage a widespread epidemic in human populations (46). For instance, hospitals were not prepared to handle a large number of patients. In addition, some countries, such as Denmark, had no models ready for disease spread in human populations that included the necessary framework to be adjusted to Sars-CoV-19 to advise the authorities from the beginning. Instead, scientists had to build these models within a very short time and develop them as data became available, without following the normal rigor in model development and validation, subjecting the model prediction to high uncertainty. Other countries, such as the UK, adapted an existing model of influenza virus spread (54) to simulate the spread of SARS-CoV-2 and advise the authorities.

## Discussion

It seems that ABMs are frequently chosen in the veterinary field to advise the authorities during outbreak situations due to their ability to incorporate a large amount of detail, while different methods are generally used for modeling infectious diseases in humans. Using a farm as the population requires much less computational power compared to modeling all people in a country, which could explain the difference in choice of method. However, because the human population is often more heterogeneously mixed and contains many more behavior patterns than livestock, ABMs would actually be a good choice of model for capturing these patterns (29). Modeling human infectious diseases on a municipality level might be sufficient to capture spatial heterogeneities and provide good tools to advise the authorities on diseases control. However, modeling on smaller aggregations than a country can create problems with parameterization due to fewer cases per subpopulation.

For convenience, some studies have categorized contacts between humans into contacts at home, work, schools, leisure, and others [e.g., 23, 24]. Precise specifications of the contacts are not defined. For instance, who are the receivers of the contacts at home, e.g., other members of the family, friends, neighbors, etc. In addition, the frequency to each of these potential receivers is sometimes not reported. The same issue exists with the other types of contacts. This limits the ability to develop ABM where exact contact structures cannot be simulated, leaving ABMs to be a more or less detailed representation of ODE models. Thus, detailed information on contact structures between individuals is essential to develop reliable predictions from ABMs.

In the veterinary field, experimental studies can be done relatively quickly to obtain necessary data to parametrize models of disease spread. This is a bigger challenge within infectious diseases of humans, as such studies would be unethical. Data sources are therefore typically limited to patients and sometime their contacts, which may include recall or selection bias, so it is highly important to rapidly initiate data collection under ongoing epidemics for the benefit of modeling future epidemics. Specifically, for SARS-CoV-2, it is often reported that cases are most infectious prior to onset of symptoms, so contact tracing of individuals should include repeated testing of contacts to ascertain the shedding of viral loads prior to the onset of symptoms.

From our own long experience in modeling disease spread and control in the veterinary field and the recent experience of modeling SARS-CoV-2 spread in Denmark, we observe that contingency and preparedness planning to handle a highly infectious disease like COVID-19 in humans has been suboptimal compared to similar preparations within the veterinary field. The importance to Denmark of livestock production and exports, including the demands for high-quality products that are made by importing countries, partly explains the importance of contingency and preparedness planning to Denmark. Nevertheless, it is unclear why contingency and preparedness planning for infectious diseases in humans has not so far been done at the same level. One potential explanation is that Denmark (in common with other developed countries) has not experienced a disease as severe as COVID-19 for many years, so contingency and preparedness plans have not been a focus of attention for the health authorities for a disease like COVID-19. We therefore recommend urgent investment in continuous development of contingency plans for human infectious diseases to develop and maintain robust models that can provide accurate predictions in case of a new outbreak with minimized prediction failures. We note that the latter has been a major discussion issue in the current epidemic (55).

## Data Availability Statement

The original contributions presented in the study are included in the article/supplementary material, further inquiries can be directed to the corresponding author/s.

## Author Contributions

TH wrote the first version of the manuscript with assistance from CK. KG, MD, and LC assisted in the writing of the manuscript and provided comments and materials. All authors read and finalized the manuscript.

## Conflict of Interest

The authors declare that the research was conducted in the absence of any commercial or financial relationships that could be construed as a potential conflict of interest.
